# Supplementary material for: Phosphatidylethanolamine Improves Postnatal Growth Retardation by Regulating Mucus Secretion of Intestinal Goblet Cells in Piglets
Source: Animals (Basel). 2024 Apr 16;14(8):1193. doi: 10.3390/ani14081193 (PMC11047706; doi:10.3390/ani14081193)
Supplement: Supplementary file 1 [file animals-14-01193-s001.zip › animals-2834235-supplementary.pdf]

Table S1. Ingredients and nutrient composition of the diets (g/kg, as-fed basis)

Item	Piglet weaning diet
Ingredients	
Corn	23.93
Extruded corn	35.00
Soybean	8.00
Fermented soybean	9.00
Whey powder	6.00
Fish meal	4.00
Plasma protein powder	2.00
Soybean oil	1.00
Glucose	3.00
Sucrose	2.00
98% *L*-lysine	0.40
DL-methionine	0.11
L-threonine	0.12
Alanine	1.59
Carrier	0.90
Organic acid calcium	0.60
Dicalcium phosphate	1.00
Choline chloride, 50%	0.01
Antioxidant	0.05
Mineral premix[1]	0.15
Vitamin premix[2]	0.04
ZnO	0.40
Acidifier	0.70
Total	100.00
Calculated chemical composition	
Digestible energy, MJ/kg	14.44
Analyzed crude protein	19.56
Apparent digestible lysine	1.14

[1] Mineral premix provided the following per kilogram of diet: Zn (ZnO), 50 mg; Cu ($CuSO_4$), 20 mg; Mn (MnO), 55 mg; Fe ($FeSO_4$), 100 mg; I (KI), 1 mg; Co ($CoSO_4$), 2 mg; Se (Na_2SeO_3), 0.3 mg.

[2] Vitamin premix provided the following per kilogram of diet: vitamin A, 8255 IU; vitamin D3, 2000 IU; vitamin E, 40 IU; vitamin B1, 2 mg; vitamin B2, 4 m g ; pantothenic acid, 15 mg; vitamin B6, 10 mg; vitamin B12, 0.05 mg; nicotinic acid, 30 mg; folic acid, 2 mg; vitamin K3, 1.5 mg; biotin, 0.2 mg; choline chloride, 800 mg; and vitamin C, 100 mg.

Table S2. Body weight of piglets at different stages

Body weight, kg	Treatments		Piglet status		SEM	*P*-value		
	CON	PE	NBW	PGR		Treatment	Status	Treatment×Status
7 day	2.33	2.35	2.79	1.88	0.06	0.874	<0.01	0.485
17day	4.64	4.61	5.51	3.73	0.12	0.877	<0.01	0.959
26 day	5.73	6.38	7.24	4.87	0.15	0.041	<0.01	0.657
33 day	6.73	7.34	8.12	5.96	0.16	0.065	0.012	0.686
40 day	8.01	9.21	9.99	7.22	0.21	<0.01	<0.01	0.992
49 day	8.26	11.48	11.24	8.50	0.31	<0.01	<0.01	0.098
